# E4 Antibodies Facilitate Detection and Type-Assignment of Active HPV Infection in Cervical Disease

**DOI:** 10.1371/journal.pone.0049974

**Published:** 2012-12-03

**Authors:** Heather Griffin, Zhonglin Wu, Rebecca Marnane, Vincent Dewar, Anco Molijn, Wim Quint, Christine Van Hoof, Frank Struyf, Brigitte Colau, David Jenkins, John Doorbar

**Affiliations:** 1 National Institute for Medical Research, London, United Kingdom; 2 GlaxoSmithKline Biologicals, Rixensart, Belgium; 3 XpePharma and Science, Wavre, Belgium; 4 DDL Diagnostic Laboratory, Voorburg, The Netherlands; National Institute of Health - National Cancer Institute, United States of America

## Abstract

High-risk human papillomavirus (HPV) infections are the cause of nearly all cases of cervical cancer. Although the detection of HPV DNA has proved useful in cervical diagnosis, it does not necessarily predict disease presence or severity, and cannot conclusively identify the causative type when multiple HPVs are present. Such limitations may be addressed using complementary approaches such as cytology, laser capture microscopy, and/or the use of infection biomarkers. One such infection biomarker is the HPV E4 protein, which is expressed at high level in cells that are supporting (or have supported) viral genome amplification. Its distribution in lesions has suggested a role in disease staging. Here we have examined whether type-specific E4 antibodies may also allow the identification and/or confirmation of causal HPV-type. To do this, type-specific polyclonal and monoclonal antibodies against three E4 proteins (HPV-16, -18, and -58) were generated and validated by ELISA and western blotting, and by immunohistochemistry (IHC) staining of epithelial rafts containing these individual HPV types. Type-specific detection of HPV and its associated disease was subsequently examined using formalin-fixed paraffin-embedded cervical intra-epithelial neoplasias (CIN, (n = 247)) and normal controls (n = 28). All koilocytotic CIN1 lesions showed type-specific E4 expression of their respective HPV types. Differences were noted amongst E4 expression patterns in CIN3. HPV-18 E4 was not detected in any of the 6 HPV-18 DNA-positive CIN3 lesions examined, whereas in HPV-16 and -58 CIN3, 28/37 (76%) and 5/9 (55.6%) expressed E4 respectively, usually in regions of epithelial differentiation. Our results demonstrate that type-specific E4 antibodies can be used to help establish causality, as may be required when multiple HPV types are detected. The unique characteristics of the E4 biomarker suggest a role in diagnosis and patient management particularly when used in combination.

## Introduction

Human papillomavirus (HPV) DNA is found in nearly all cases of cervical cancer (>99.7%) and high-grade pre-cancers [1], and has been used to assign causality to a HPV type in a lesion. Despite the widespread utility of the approach, genotyping alone does not allow HPV-induced disease to be distinguished from HPV-associated latent or asymptomatic infections (where HPV DNA is present in the absence of disease), and cannot always discriminate active infections (where HPV DNA is present and causative of disease) from the presence of passive viral particles that may be found at the epithelial surface. In particular, genotyping alone cannot reliably identify the causative HPV type when multiple infections are present in a lesion, and in recent years, such limitations have prompted the development of complementary methodologies. To a large extent, such studies have moved from the analysis of HPV DNA alone, to the analysis of markers of active viral infection, such as viral transcripts, viral proteins [2], and/or cellular gene products that can be used as surrogate markers of viral E6/E7 gene activity, such as p16 [3] and/or minichromosome maintenance protein (MCM) [4]. Although these approaches have considerable potential, they generally have limited ability to distinguish HPV type, and/or are difficult to use on standard formalin fixed paraffin-embedded (FFPE) tissue where RNA degradation may have occurred. As such, they have not yet been widely applied to the problem of assigning causality or confirming causality when multiple HPV types are found.

The viral E4 protein is abundantly expressed in infections caused by diverse HPV types, and as a viral biomarker, it can identify cells supporting vegetative viral genome amplification and virus assembly (cells supporting genome amplification always express E4 [5]). In the upper layers of the epithelium, the E4 protein assembles into stable amyloid-like fibres and accumulates in the lesion to varying extents depending on lesion grade [6], [7]. Its great abundance makes it simple to detect in biopsy material, while the sequence diversity between E4s of different type suggests that E4 antibodies may be useful in establishing (or confirming) causality. These characteristics make E4 a promising biomarker of active HPV infection, perhaps in conjunction with surrogate markers of the viral E6/E7 oncogenes such as MCM or p16, which can also mark undifferentiated high-grade lesions where E4 expression may be absent [6], [7].

Here we have examined this hypothesis by generating type-specific antibodies to the E4 proteins of HPV-16, -18 and -58, and show that these reagents can be used to visualize type-specific E4 expression in FFPE clinical biopsies by immuno-histochemistry (IHC). The primary aim of the study was to establish a simple method for confirming HPV causality, as is required (for instance) when assessing vaccine efficacy. To do this, type-specific staining was carried out on 275 cervical biopsy specimens (comprising 247 CIN (cervical intra-epithelial neoplasia) and 28 normal cervical tissues)) of different disease grades and different HPV association in order to demonstrate the general utility of the approach (76 of which are described in detail in Table 1). The study supports our previous suggestion for a role of the E4 biomarker in diagnosis and disease-staging, and extends the E4 approach to cover the confirmation of HPV causality.

**Table 1:** Immuno-histochemistry results with type-specific anti-E4 antibodies on cervical biopsies.

	Detection of E4 by anti-E4 antibodies					Pathology	HPV PCR DNA Typing by WTS-PCR	HPV LCM-PCR result on lesional area (if available)£
	cross-reactive		type-specific					
Biopsy	E4 HPV-16, 31, 35 (TVG405 regime 1)	E4 HPV-16, 31, 35, & 18 and 45 (TVG405 regime 2)	E4 HPV-16 (MoAb16E4_35–42_)	E4 HPV-18 (R18E4_53–60_)	E4 HPV-58 (R58E4_23–30_)			
1	n/a	–	−	n/a	n/a	Normal	–	n/a
2	n/a	n/a	−	−	n/a	Normal	–	n/a
3	n/a	n/a	−	−	n/a	Normal	–	n/a
4	n/a	–	−	−	n/a	Borderline (CIN1)	18	–
5	n/a	–	n/a	n/a	+*	Borderline (CIN1)	18,31,58,66	–
6	n/a	n/a	−	−	n/a	CIN1	39	39
7	n/a	n/a	−	−	n/a	CIN1	33	33
8	n/a	n/a	−	−	n/a	CIN1	–	n/a
9	n/a	n/a	+	−	n/a	CIN1	6,16	16
10	−	n/a	−	−	n/a	CIN1	(16),52	52
11	n/a	+	n/a	+	+	CIN1	18,58	18,58
12	n/a	+	n/a	+	n/a	CIN1	18,66	18,66
13	n/a	n/a	+	−	n/a	CIN1	16	n/a
14	n/a	+	−	+	n/a	CIN1	18,51,52	n/a
15	n/a	n/a	+	−	n/a	CIN1	16	16
16	n/a	n/a	−	−	n/a	CIN1	35,52	n/a
17	n/a	+	−	+	n/a	CIN1	18	18,43
18	n/a	+	−	+	n/a	CIN1	18	n/a
19	n/a	−	n/a	−	n/a	CIN1/2	18,31	31**
20	n/a	−	−***	−***	n/a	CIN2	16	16
21	n/a	n/a	−	−	n/a	CIN2	35	n/a
22	n/a	n/a	−	−	n/a	CIN2	51	34, 51
23	n/a	n/a	−	−	n/a	CIN2	16,68	16
24	n/a	n/a	−***	−***	n/a	CIN2	16	16
25	n/a	n/a	−	−	n/a	CIN2	16,51	16,51
26	n/a	n/a	−	−	n/a	CIN2	52	n/a
27	+	n/a	+	−	n/a	CIN2	16,51	16
28	−	n/a	−	−	n/a	CIN2	16,31,33	31
29	n/a	n/a	−	−	n/a	CIN2	16,31,33,51	51
30	n/a	n/a	n/a	−	+	CIN2	18,58	58
31	+	n/a	+	−	n/a	CIN2	16	16
32	−***	n/a	−***	−***	n/a	CIN2	16,51	16
33	n/a	n/a	+	n/a	n/a	CIN2	16	16
34	n/a	n/a	−	−	n/a	CIN2	58	n/a
35	+	n/a	+	n/a	−	CIN2	16	16
36	n/a	n/a	−	n/a	+	CIN2	16,58	58
37	+	n/a	+	−	n/a	CIN2	16	16
38	n/a	+	−	+	n/a	CIN2	18,54	18
39	n/a	+	n/a	+	n/a	CIN2	18	18
40	n/a	+	−	+	n/a	CIN2	18	18
41	n/a	n/a	+	−	n/a	CIN2	16,18	16
42	n/a	n/a	+	−	n/a	CIN2	16,51, 52	16
43	+	n/a	+	−	n/a	CIN2	16/70	16/70
44	+	n/a	+	−	−	CIN2	16/31	n/a
45	+	n/a	+	n/a	−	CIN2	16	n/a
46	n/a	n/a	+	−	n/a	CIN2	16	16
47	n/a	n/a	−	−	n/a	CIN2	18, 52	16
48	n/a	n/a	−	−	+	CIN2	16/39/51/58	18/58
49	+	n/a	+	−	−	CIN2/3	16/18/31	16
50	n/a	n/a	+	−	n/a	CIN3	16	16
51	n/a	n/a	+	−	n/a	CIN3	16	16
52	n/a	n/a	−	−	n/a	CIN3	16	16
53	n/a	−	−	n/a	n/a	CIN3	16	16
54	n/a	n/a	−	−	n/a	CIN3	16,51	16
55	+	n/a	+	−	n/a	CIN3	16	16
56	n/a	n/a	n/a	−	n/a	CIN3	18	n/a
57	n/a	−	n/a	−	n/a	CIN3	18	n/a
58	n/a	−	n/a	−	n/a	CIN3	18	n/a
59	n/a	n/a	+	−	n/a	CIN3	16	16
60	n/a	n/a	−	n/a	+	CIN3	58	n/a
61	−	n/a	−	−	n/a	CIN3	16/18	16/18
62	n/a	n/a	−	n/a	+	CIN3	58	58
63	n/a	n/a	n/a	n/a	+	CIN3	58	n/a
64	n/a	n/a	−	n/a	+	CIN3	58	58
65	n/a	n/a	+	−	n/a	CIN3	16,18	16
66	n/a	n/a	+	−	n/a	CIN3	16	16,52
67	n/a	−	n/a	−	n/a	CIN3	18	n/a
68	n/a	n/a	n/a	n/a	−	CIN3	58	58
69	n/a	n/a	−	n/a	−	CIN3	58	58
70	n/a	n/a	−	−	n/a	CIN3	16	16
71	n/a	n/a	+	−	n/a	CIN3	16,52	16
72	n/a	n/a	−	−	n/a	CIN3	16	16
73	n/a	n/a	−	n/a	−	CIN3	58	n/a
74	n/a	n/a	−	n/a	−	CIN3	58	n/a
75	n/a	n/a	+	n/a	n/a	CIN3	16	16
76	−	n/a	−	n/a	+	CIN3	16/52/58	58

HPV-16, HPV-18, and HPV-58 containing raft controls were positive with the appropriate anti-E4 antibodies in each experiment.

− = negative;

+ = positive.

WTS-PCR = whole tissue section PCR.

N/A = Not applicable (Tissues section not tested).

* 58 positive area is different to the area sampled by LCM (laser capture micro-dissection).

** 31 positive area lost from slide during immunostaining protocol.

*** differentiated layers lost from slide during immunostaining protocol.

() = weakly positive for this type.

CIN: cervical intraepithelial neoplasia.

£ All HPV types were detected by LCM-PCR as single type HPV infections in different CIN lesion areas.

## Materials and Methods

### Ethical Statement

The studies complied with the Helsinki Declaration of 1975, as revised in 1983. Appropriate ethical review committees approved three studies and informed written consent was obtained for all analyses described in this manuscript. Individual Ethical Review Boards included: University of New Mexico, Albuquerque, US; University of Texas, Houston, US; Marshfield Clinic, Marshfield, US; University of California, San Francisco, US; San Francisco General Hospital, San Francisco, US; Dartmouth Medical Centre, Labanon, US; Morristown Memorial Hospital, Morristown, US; University of Louisville, Louisville, US; University of Georgia, Augusta, US; Thomas Jefferson University, Philadelphia, US; Quorum Review IRB, Seattle, US; Optimum Clinical Research, Oshawa, Canada; University of Manitoba, Winnipeg, US; University of Alberta, Edmonton, Canada; Hospital de Clinicas de Porto Alegre, Rio Grande do Sul, Brazil; Comitê de Ética em Pesquisa da Secretaria Municipal de Saúde de São Paulo, São Paulo, Brazil; Comitê de Ética em Pesquisa em Seres Humanos do Hospital de Clinicas, Paraná, Brazil; Comitê de Ética em Pesquisa da Faculdade de Ciências Medicas, São Paulo, Brazil; Hospital Universitário Walter Cantídio da Universidade Federal do Ceará –COMEPE, Ceará Brazil. The study was registered on ClinicalTrials.gov with number NCT00120848.

### Animals

Six-week old female BALB/c mice (Harlan, Netherlands) and 10–12 week old New Zealand white rabbits (Eurogentec, Belgium) were cared for in accordance with local and international animal welfare regulations and guidelines.

### Selection of E4 Peptides and the Generation of HPV Type-Specific Antibodies

To prepare antibodies that can specifically identify the E4 proteins of HPV-16, -18 and -58, sequence alignments were first carried out, and short peptide sequences (8 to 9 amino acids in length) were chosen in regions of highest divergence. These peptides were synthesised chemically before being used as antigens to generate anti-peptide polyclonal antibodies (28 day immunization protocol carried out by Eurogentec). For the anti-HPV-18 E4 polyclonal antibody (R18E4_53–60_), rabbits were injected on Days 0, 7, 10 and 18 with an 8 amino acid peptide, (DSRRSSIV), conjugated using glutaraldehyde to keyhole limpet hemocyanin (KLH). For the anti-HPV-58 E4 polyclonal antibody (R58E4_23–30_), rabbits were immunized using the same schedule, but with a nona-peptide (CTTKVHRGQ) containing an N-terminal cysteine residue that was conjugated to KLH using m-maleimidobenzoil-N-hydroxysuccinimide ester (MBS). Bleeding was performed on day 28. The rabbit polyclonal anti-HPV E4 antibody (RE4) was raised against the full length E4 protein prepared as a HPV18 maltose binding protein fusion (MBP-E4). The anti-HPV-16 E4 monoclonal (MoAb16E4_35–42_) and polyclonal (M16E4_35–42_) antibodies were prepared using a nona-peptide, CAPKKHRRL containing an N-terminal cysteine residue that was conjugated to ovalbumin using MBS. Female BALB/c mice were immunized 4 times subcutaneously in a 13-day period with conjugate combined with the GlaxoSmithKline (GSK) Biologicals Adjuvant System AS02_A_ containing MPL (3-*O*-desacyl-4′- monophosphoryl lipid A; GSK) and QS21 (*Quillaja saponaria* Molina, fraction 21; Antigenics, New York, NY, USA) in an o/w emulsion [8]. Hybridoma cell lines were subsequently generated by fusion of lymph node cells from immunized mice with mouse myeloma cell line SP2/0, using 50% polyethylene glycol 1500 (Roche, Basel, Switzerland).

### Purification of Recombinant Maltose-binding Protein-E4 Fusion Proteins

Maltose binding protein (MBP)-E4 fusion proteins were produced according to the manufacturer’s instructions (New England Biolabs, Beverly, MA, USA).

### Western Blotting and Enzyme-linked Immunosorbent Assay (ELISA)

(1.25 µg) purified recombinant MBP-E4 fusion proteins were denatured and run on a 10% NuPAGE™ Bis-Tris gel according to the manufacturer’s instructions (Invitrogen, Paisley, UK). Proteins were then transferred to a polyvinylidene-difluoride membrane and detected using rabbit sera or monoclonal antibodies and standard protocols [9]. Membranes were probed with anti-MBP antibodies (New England Biolabs) to control for expression of different MBP-E4 fusion proteins. Standard ELISA was used to screen hybridomas for specific monoclonal anti-peptide antibodies (plate coated with 2 µg/ml purified peptide in PBS) and anti-protein antibodies (plate coated with 0.5 µg/ml purified recombinant MBP-E4 fusion protein in PBS).

### Raft Culture

The NIKS cell line (Stratatech Corporation, Madison, WI, USA) was cultured in the presence of J2 3T3 fibroblast feeders which were maintained at low passage in selected growth media. The transfection of NIKS cells, and the generation of stratified squamous epithelial rafts were performed according to Lambert *et al.* [10]. The presence of episomal HPV-16, HPV-18, or HPV-58 genomes in blasticidin-resistant sub-clones was confirmed by polymerase chain reaction (PCR) and Southern blotting. Histological sections (5 µm) of rafts fixed in buffered formalin and embedded in paraffin were used for IHC.

### Clinical Samples

Colposcopic biopsies of CIN2 and 3 were mostly from HERACLES (EPI-108290), a GSK-funded retrospective, cross-sectional, European multicentre epidemiological study on HPV type distribution in women with CIN2 and 3 [11]. Biopsies with CIN1 were obtained from HPV-007 (NCT00120848), a phase IIb follow-up study of the efficacy of the GSK Biologicals HPV-16/18 L1 VLP AS04 vaccine (*Cervarix*™) [12]. The majority of the normal cervical biopsies came from the anonymous collection held at NIMR, London, with patient data for all the samples being anonymized. All biopsies were fixed in buffered formalin and embedded in paraffin.

### Pathological Diagnosis and Grading

The diagnosis and grading of areas of CIN1, 2 and 3 were made at Quest Diagnostics (Teterboro, NJ, USA) according to standard criteria on the haematoxylin and eosin (H&E) -stained sections [13] by majority diagnosis of three expert pathologists. p16 IHC was used to support diagnosis in HPV-007 [14]. The study clinical diagnosis was the worst grade of CIN represented. Biopsies with CIN2 and 3 might include lower grade abnormalities and normal cervical epithelium. CIN1 was defined as cases with classical koilocytotic CIN1. Borderline CIN1 included squamous epithelium showing changes suggestive of CIN1 without definite koilocytosis and atypical immature metaplasia [15]. In Table 1 there was one biopsy where CIN1 and 2 was recorded (biopsy 19) whereas another biopsy where CIN2 and 3 was determined (biopsy 49) by pathologists. CIN grading was re-assessed by two independent expert pathologists at DDL (Voorburg, The Netherlands) on the section used for E4 IHC. No discrepancies were found amongst the independent pathologists at DDL. On a number of occasions however, we found that the disease area was no longer present in the tissue section under analysis, usually because it was small and because we had through the lesion and into normal tissue. In general, diagnosis was confirmed by four or five pathologists using common criteria.

All biopsy blocks were sectioned according to the sandwich cutting procedure, which ensured that PCR for HPV and E4 IHC were performed within a sandwich of histology diagnosis as described previously [16].

### Immunohistochemical Staining (IHC)

HPV E4, MCM and L1 IHC was performed on raft or cervical biopsy sections according to standard procedures [7]. For epitope retrieval, slides were incubated in solution D pH 9.0 (Dako, Glostrup, Denmark) for 10 min at room temperature prior to autoclaving for 2 min at 121°C. The primary HPV type-specific anti-E4 antibodies were diluted 50-fold (MoAb16E4_35–42_; concentrated supernatant, 2,111 mg/ml) or 100-fold (M16E4_35–42_, R58E4_23–30_, R18E4_53–60_). HPV anti-L1 (BD Pharmingen, Oxford, UK) and anti-MCM antibody (Abcam, Cambridge, UK) were used on some sections and were diluted 100-fold, while RE4 non-HPV type-specific antibodies (raised against the whole E4 protein) [17] were diluted 250-fold before use. All detections except for MoAb16E4 _35–42_ were carried out using anti-rabbit or anti-mouse biotinylated antibody (dilution, 1∶150, Vector, Peterborough, UK) followed by development using ABC kit (Vector) and TSA-reagent (In red, PerkinElmer, Boston, USA).

Due to limited number of sections for each case (usually one or two), slides used for E4 IHC with anti-HPV-18 E4 R18E4_53–60_ or anti-HPV-58 E4 R58E4_23–30_ rabbit polyclonal antibodies were subsequently used for E4 IHC with the anti-HPV-16 E4 MoAb16E4_35–42_ mouse monoclonal antibody, followed by visualisation with a 150-fold diluted Alexa-488 (green) conjugated anti-mouse secondary antibody (Invitrogen). When sufficient sections were available for staining, tissue sections were also stained for E4 as a positive poly-reactive control using the previously developed anti-HPV E4 antibody (human Fab TVG405) [17] diluted 150-fold, and incubated for one hour which allowed detection of HPV-16, 31 or 35 E4 (staining regime 1). Overnight incubation allowed detection all E4 proteins recognized by this antibody (HPV-16, 31, 35 18 or 45). This is referred to as staining regime 2. Nuclear counterstain was performed with 4′-6-diamidino-2-phenylindole (DAPI, 1 mg/ml 200- to 500-fold diluted, Sigma, St-Louis, MO, USA) before mounting in Citifluor medium (Agar Scientific, Essex, UK) for fluorescence microscopy (Nikon, EFD-3).

The IHC staining and reading were done by three individuals (HG, ZW, and DJ) who where blind to both the HPVDNA data and the CIN diagnosis. Persons who did not work in the lab made decision of which antibodies to use for each case. The E4 staining pattern was very distinctive and intense, and in this study no disagreements were encountered with regard to scoring the presence or absence of the E4 protein in the lesion. Images were captured using an Axiovision microscope system (Zeiss).

### HPV DNA Detection and Laser Capture Micro-dissection

HPV DNA genotyping was done according to the PCR algorithm described earlier [18]. The highly sensitive broad spectrum short PCR fragment (SPF_10_) PCR-DNA ELISA (DEIA) immunoassay system was used for both whole tissue section (WTS) and laser capture micro-dissection (LCM) PCR [19], [20], combined with the reverse hybridisation Line Probe Assay (LiPA_25_) version 1 HPV genotyping system (Labo Biomedical Products, Rijswijk, The Netherlands based on licensed Innogenetics SPF_10_ Technology), which identifies 25 different HPV genotypes, 14 high-risk HPV types (HPV-16, 18, 31, 33, 35, 39, 45, 51, 52, 56, 58, 59, 66, 68) and 11 low-risk HPV types (HPV 6, 11, 34, 40, 42, 43, 44, 53, 54, 70, 74). For available specimens with multiple HPV types as determined by WTS-PCR, LCM-PCR was performed as recently described [16] usually on a separate section from that for E4 IHC. WTS-PCR was performed on all cases examined in this study for the identification of HPV type(s) present in a lesion. In some cases LCM-PCR was also performed, and in these instances the results were used for the final HPV type assignment and for comparison with IHC.

## Results

### Generation of Polyclonal and Monoclonal Antibodies to E4

**Figure 1:**
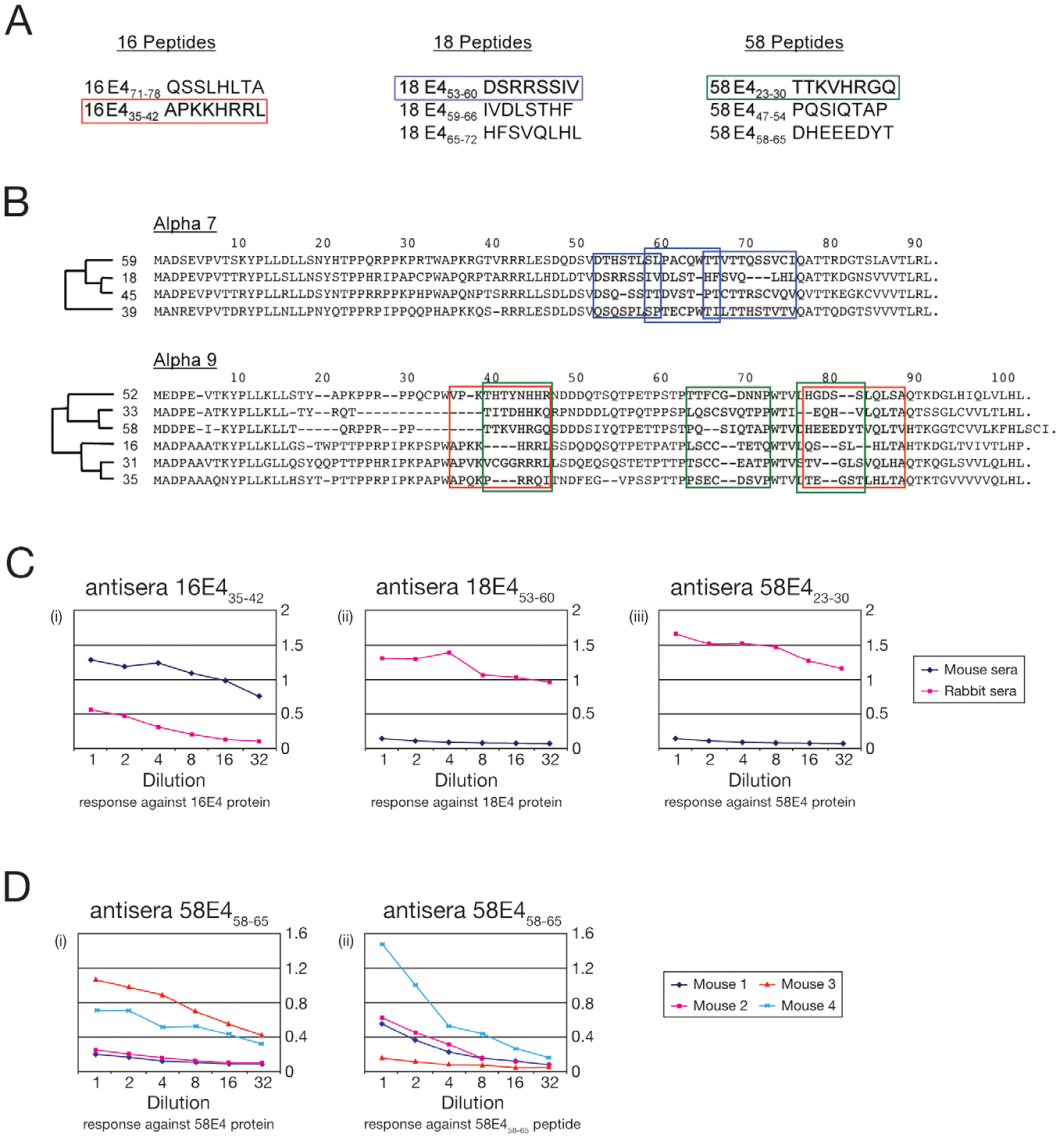
Selection and evaluation of immunogens used for the production of HPV type-specific anti-E4 antibodies. A) All of the target peptides that were used as immunogens in this study are listed along with their amino acid positions within E4. The peptides that gave rise to type-specific E4 antibodies are boxed. B) The phylogenetic relationship and amino acid sequence alignment of the 10 HPV E4 proteins used to evaluate antibody type-specificity are shown. All of the selected peptide sequences differed from sequences found in other E4 proteins by at least 5 amino acids. Red, blue and green boxes encompass the HPV-16, -18 and -58 E4 peptides, respectively. C) ELISA results comparing the mice and rabbit polyclonal antibody responses against the full length E4 proteins of HPV-16, -18 or -58 following immunization with, (i) peptide 16E4_35–42_, (ii) peptide 58E4_23–30_ and (iii) peptide 18E4_53–60_ (as indicated below the graphs). Antibodies from rabbits and mice showed dramatically different characteristics, even when the same immunogen was used. D) ELISA results comparing the different responses to the same injected peptide (58E4_58–65_) in four inbred BALB/c mice. Reactivity against the peptide immunogen (58E4_58–65_) is shown in (i) on the left, with the corresponding response to the full-length 58E4 protein (ii) is shown on the right.

All the peptides from divergent regions of E4 proteins of HPV-16, -18 and -58 (see Fig. 1A and 1B) were immunogenic, with several stimulating production of antibodies that reacted well against the full-length E4 proteins. From these, the M16E4_35–42_ mouse polyclonal (and subsequently MoAb16E4_35–42_ mouse monoclonal antibodies) raised against the HPV-16 E4_35–42_ peptide, the R18E4_53–60_ rabbit polyclonal antibody against the HPV-18 E4_53–60_ peptide, and the R58E4_23–30_ rabbit polyclonal antibody against the HPV-58 E4_23–30_ peptide yielded highly potent ELISA responses (Fig. 1C) against their respective full-length E4 proteins, and were chosen for further analysis. The anti-peptide response did not always predict a good anti-protein response with widely different responses even amongst genetically identical inbred mice. This can be seen with peptide 58E4_58–65_ (Fig. 1D), where the animal (mouse 3) showing the weakest anti-peptide response in ELISA showed the best response to the full-length protein. Peptides 16E4_35–42_, 18E4_53–60_, 58E4_23–30_ gave rise to potent and reproducible anti-peptide and anti-protein immune responses in mice (16E4_35–42_) or rabbits (18E4_53–60_, 58E4_23–30_). A useful anti-protein response could only be assessed for a particular peptide following immunization of both species.

### Specificity of the Newly Generated Anti-E4 antibodies in Western Blot and ELISA

**Figure 2:**
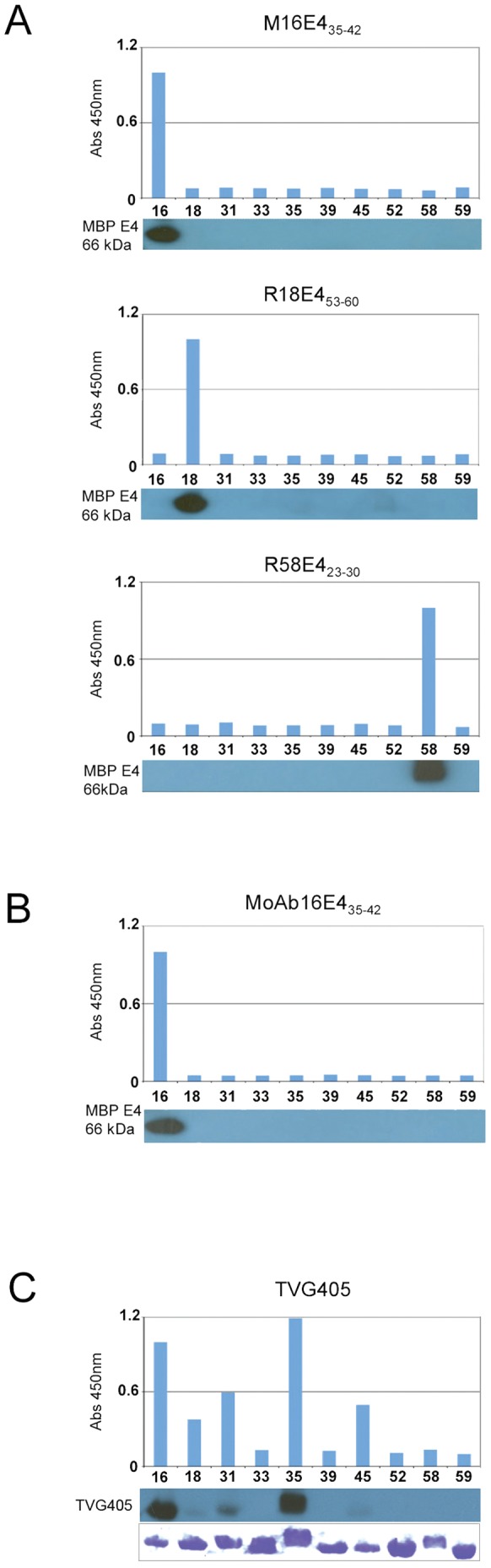
Specificity of HPV type-specific antibodies against different HPV E1?E4 proteins by ELISA and Western blotting. Optical density measurements from ELISA on a panel of 10 recombinant maltose-binding E4 proteins (HPV-16, 18, 31, 33, 35, 39, 45, 52, 58, and 59) used to evaluate the specificity of on M16E4_35–42_, R18E4_53–60_ and R58E4_23–30_ polyclonal antibodies (A) and MoAb16E4_35–42_ monoclonal antibody (B). Cross-reactive TVG405 was used for comparison (C) and the relative abundance of the various MBP proteins is shown following staining with Coomassie blue (lower panel of C). Western blot results are shown as inserts under the corresponding graphs presenting the ELISA results.

Specificity of the newly generated HPV type-specific anti-E4 antibodies (MoAb16E4_35–42_, M16E4_35–42_, R58E4_23–30_, and R18E4_53–60_) was assessed by testing cross-reactivity with MBP-E4 fusion proteins prepared from a panel of 10 different HPV types (HPV-16, -18, -31, -33, -35, -39, -45, -52, -58, and -59) by ELISA and western blotting. As shown in Fig. 2A and 2B, the MoAb16E4_35–42_ monoclonal and M16E4_35–42_ polyclonal antibodies, the R18E4_53–60_ polyclonal antibody, and the R58E4_23–30_ polyclonal antibody, were highly specific for HPV-16, 18, and 58 E4 proteins respectively in both ELISA and western blot analyses, as predicted by sequence alignment (Fig. 1B). The previously identified monoclonal TVG405 poly-reactive antibody, which detects five types of HPV E4 protein including those of HPV-16, -18, -31, -35 and -45, was also tested alongside as a control (Fig. 2C).

### Specificity of the Newly Generated Anti-E4 Antibodies in Organotypic Raft Cultures

**Figure 3:**
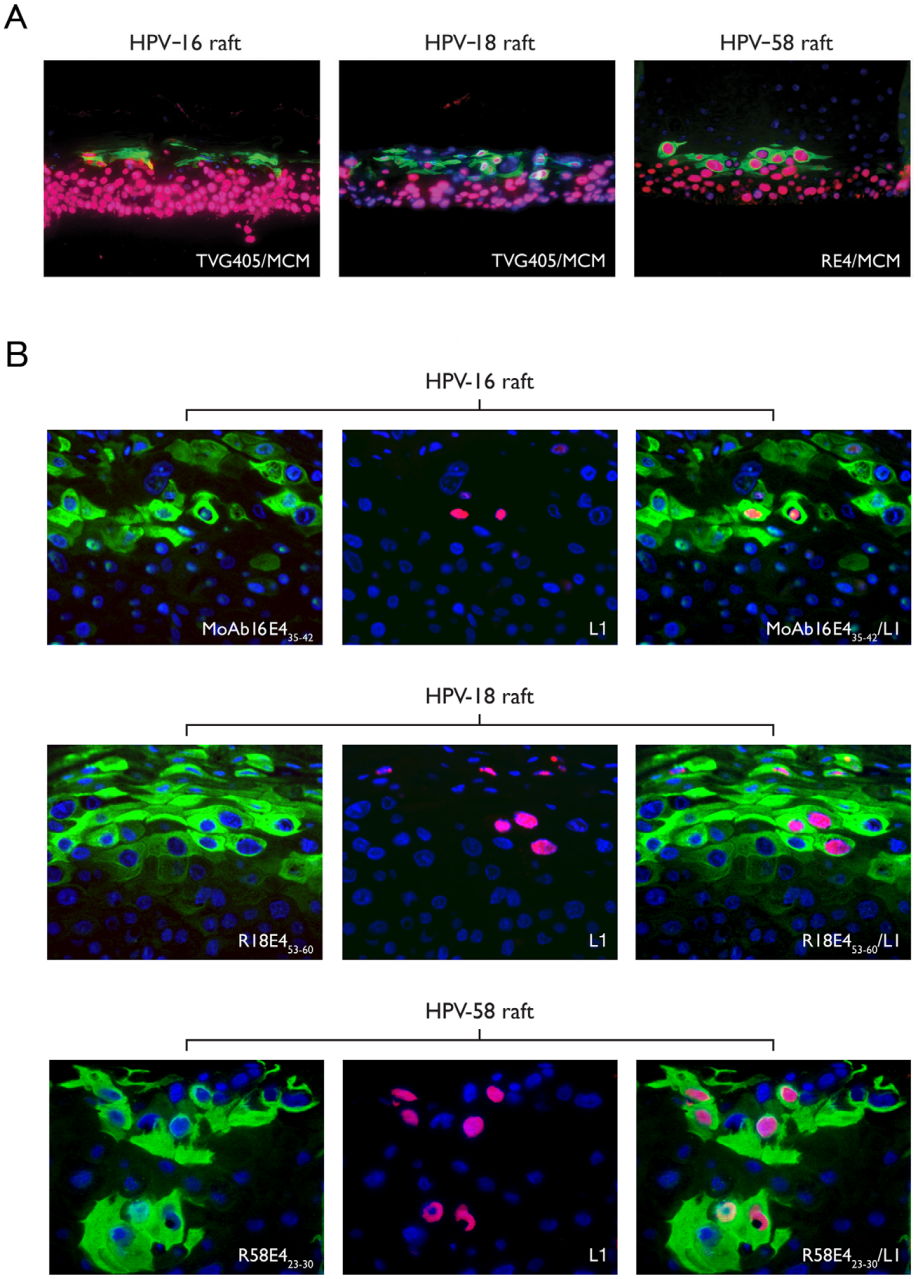
Evaluation of E4, MCM and L1 protein expression in HPV16, 18 and 58 rafts. (A) HPV-16 and 18 rafts were probed with cross-reactive TVG405 (green) and MCM (red) antibodies. The HPV-58 raft was stained with cross-reactive (RE4) rabbit sera (green) and MCM (red) antibody. The staining patterns are typical of those expected for high-risk HPV types. (B) Novel HPV-58 rafts were further probed with R58E4_23–30_ (green) and HPV L1 (red) antibodies and compared with rafts containing HPV16 and 18 and stained with HPV L1 and MoAb16E4_35–42_ and R18E4_53–60_ respectively. The detection of L1 in a subset of the E4-positive cells was seen in each raft. All sections were counterstained with 4′,6′-diamino-2-diamino-2-phenylindole (DAPI, blue). The images were taken on a microscope using a 10x (A) or 40x (B) objective. The merged image (E4 green/MCM red) is shown on the right of the figure. L1 was detected in the superficial and mid-spinous cell layersp.

**Figure 4:**
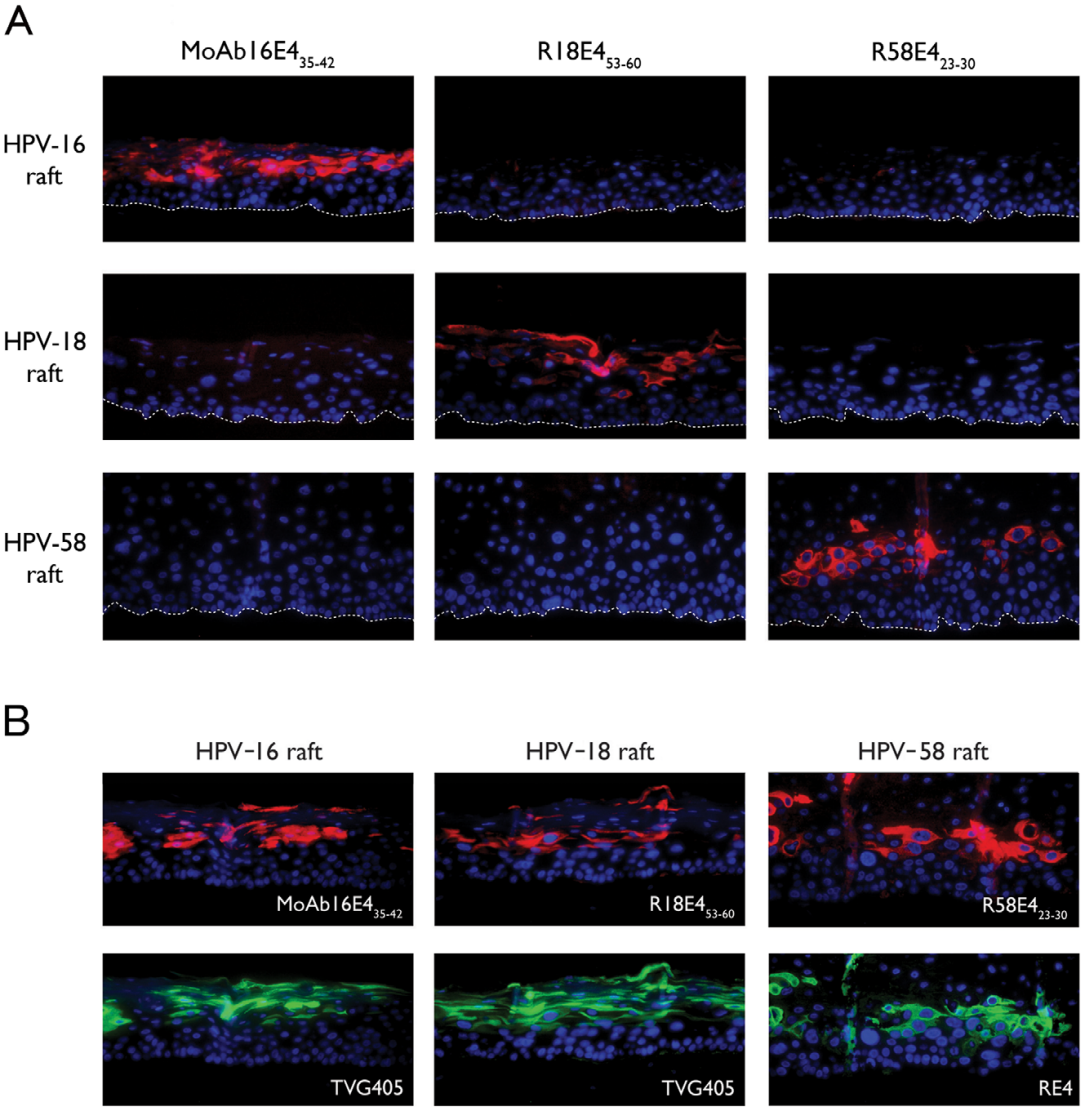
Evaluation of antibody specificity using rafts containing HPV-16, 18 and 58. A) Raft sections containing HPV-16, -18 or -58 genomes were individually probed with MoAb16E4_35–42_, R18E4_53–60_ and R58E4_23–30_ antibodies (red) and were counterstained with DAPI (blue). The different antibodies allowed type-specific detection of E4 and showed no cross-reactivity amongst the types tested. B) E4 protein expression was detected in HPV-16, -18 and -58 rafts after pre-treatment with solution D, pH 9.0 and autoclaved for 2 min, prior to incubation with MoAb16E4_35–42_, R18E4_53–60_ and R58E4_23–30_ antibodies (red - upper panels). In the lower panels, sections were pre-treated in the same way prior to incubation with cross-reacting TVG405 or RE4 (green). All sections were counterstained with 4′,6′-diamino-2-diamino-2-phenylindole (DAPI, blue).

Differentiating epithelial rafts from NIKS cell-lines maintaining episomal HPV-16, -18 or -58 were used to demonstrate specificity of E4 detection by IHC. The typical E4 expression pattern [5], [7] defined by the poly-reactive antibodies was also apparent using the type-specific antibodies in rafts containing each HPV type individually, and was characteristic of what is seen *in vivo* in low-grade disease (Fig. 3A). Antibodies to HPV L1 confirmed the presence of viral capsid proteins in the upper-most differentiated layers of the HPV-16, -18 and -58 rafts (Fig. 3B). The HPV 18 rafts were analysed in more detail by electron microscopy, and revealed the presence of virus arrays in the nucleus. The newly generated HPV type-specific anti-E4 antibodies (M16E4_35–42_, MoAb16E4_35–42_, R18E4_53–60_ and R58E4_23–30_) did not cross-react with the other types of HPV E4 tested (see Fig. 4A). In the HPV-16 or 18-infected rafts, MoAb16E4_35–42_ (M16E4_35–42_ data not shown) and R18E4_53–60_ produced similar patterns of staining as the TVG405 anti-E4 Ab (Fig. 4B). The same pattern was also seen for HPV-58 with R58E4_23–30_ and a non-HPV type-specific HPV E4 rabbit polyclonal antibody (RE4 (see Fig. 4B)).

### Optimisation of E4 Protein Detection by IHC in Biopsy Sections with the Newly Generated Anti-E4 Antibodies

Six different epitope retrieval solutions were tested with two different heating methods (12 min microwave or 2 min autoclave) to optimise staining. The newly generated reagents (M16E4_35–42,_ MoAb16E4_35–42_, R58E4_23–30_, and R18E4_53–60_) were compared with TVG405 as well as with the RE4 non-HPV type-specific antibodies [7]. Autoclave treatment was consistently more effective than microwave treatment, and pH-9.0 buffers containing EDTA out-performed those at low pH (data not shown). One epitope retrieval regime (solution D pH 9.0 (Dako) combined with autoclaving for 2 min) was particularly effective, allowing strong staining with all HPV type-specific anti-E4 antibodies tested (Fig. 4B). A common epitope exposure procedure for HPV type-specific detection of all three E4 proteins paves the way for using these antibodies in diagnosis.

### HPV Type-specific E4 Detection by IHC on Cervical Biopsy Sections

In Table 1 we present data from 76 representative cervical biopsies, 71 of which were unambiguously classified as CIN. 43 biopsies contained a single HPV DNA type, with 29 biopsies containing multiple HPV types as determined by WTS-PCR. Three histologically normal biopsies and one CIN1 biopsy shown in the table were negative for HPV DNA by WTS-PCR. 55 of these biopsy sections were also available for LCM-PCR. Apart from biopsies 4 and 5, which were borderline CIN1 and HPV DNA-negative by LCM-PCR, the different lesional areas in multiply-infected CIN were assigned to individual HPV types by LCM-PCR (see also [16]).

The results of IHC staining with the HPV type-specific anti-E4 antibodies were compared with the HPV DNA type assignment by WTS-PCR, and when available, by LCM-PCR and with TVG405 immunostaining (Table 1).

**Figure 5:**
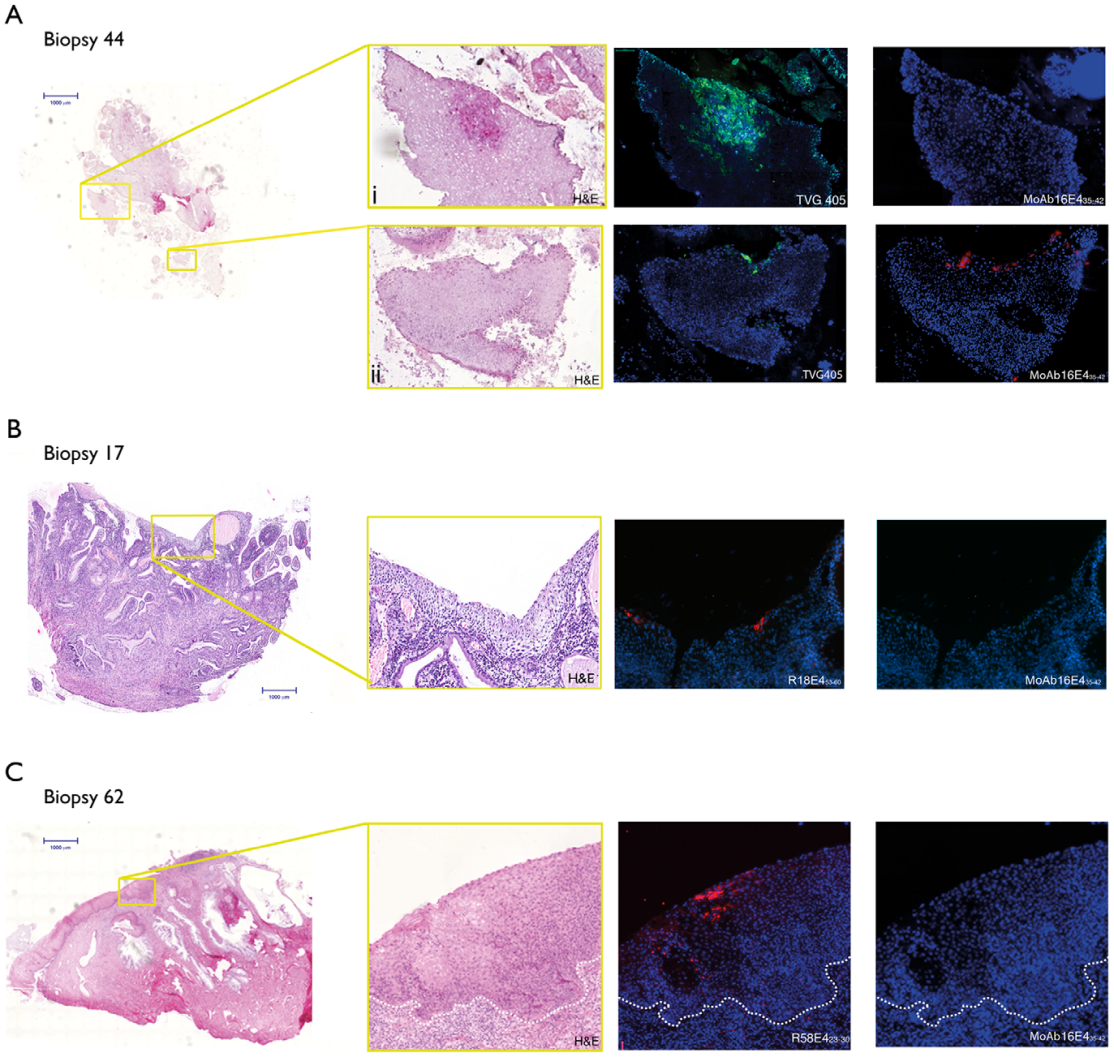
Immunohistochemical staining of HPV E4 proteins in productive cervical lesions caused by different HPV types using MoAb16E4_35–42_, R18E4_53–60_, R58E4_23–30_ or TVG405 antibodies. A) Scan of hematoxylin and eosin (H&E) stained biopsy 44 (genotype HPV-16, 31 by WTS-PCR) with areas of interest boxed in yellow. Detection of HPV-31 E4 in region of CIN1 (i) using TVG 405; MoAb16E4_35–42_ antibody gave no staining on the same tissue section. HPV-16 E4 is detected using MoAb16E4_35–42_ antibody in a region of CIN2 (ii) and confirmed using TVG405 on the same tissue section. B) Scan of H&E stained biopsy 62 (genotype HPV-58 by WTS-PCR and LCM-PCR) with area of interest boxed in yellow. Detection of HPV-58 E4 protein expression by R58E4_23–30_ antibody in an HPV-58-infected region classified as CIN2. MoAb16E4_35–42_ antibody gave no staining on the same tissue section indicating no cross-reactivity. C) Scan of H&E stained section biopsy 16 (genotype HPV-18 by WTS-PCR) with area of interest boxed in yellow. Detection of HPV-18 E4 protein expression using R18E4_53–60_ antibody in an HPV-18-infected CIN1 lesion and confirmation by TVG405 staining regime 2 on the same tissue section. MoAb16E4_35–42_ antibody gave no staining indicating no cross-reactivity. All sections were counterstained with 4′,6′-diamino-2-diamino-2-phenylindole (DAPI, blue).

MoAb16E4_35–42_ detected HPV-16 E4 protein expression in 3/4 CIN1 that contained HPV16 according to WTS-PCR. In biopsy 10, that was E4 negative, both HPV-16 and -52 DNA were detected by WTS-PCR, but only HPV-52 DNA was identified by LCM-PCR, therefore HPV-52 was assigned as the causative type. Three of the HPV-16 CIN2 typed by WTS-PCR (out of a total of 19 shown) were damaged during the IHC process. In biopsy 41, HPV-16 and -18 were detected by WTS-PCR but only HPV-16 was identified by LCM-PCR in the lesional area. HPV16 E4 was also detected in this region, providing unambiguous confirmation that this HPV type was active in driving the CIN2-grade abnormality. The CIN2/3 and 8 of the CIN3 lesions assigned by WTS-PCR to HPV 16 DNA (total 15 in Table 1) were E4 positive by IHC. The LCM-PCR results were in total agreement with the E4 IHC, and revealed the presence of other types in 5 biopsies (biopsy 10, 28, 29, 36 and 76), which were shown to be negative for HPV16 E4. No other sections stained positive with the type-specific HPV-16 antibody, including lesions containing HPV-31 (biopsy 28), -35 (biopsy 21), -51(biopsy 22), -52 (biopsy 26) and -58 (biopsy 34) which are phylogenetically related to HPV-16, and which are also members of the alpha 9 species [21]. Seven HPV-16-typed biopsies were also stained positively with the poly-specific TVG405 antibody in accordance with the type-specific stains. Biopsy 44 contained HPV-16 and -31 by WTS-PCR and was positively stained with TVG405 (Fig. 5A(i)) in one region but not with MoAb16E4_35–42,_ indicating an HPV31 infection in this lesional area. In contrast, in another region of biopsy 44, positive staining was shown by both antibodies suggesting an active HPV16 infection (rather than 31) in this region (Fig. 5A(ii)).

The antibody R18E4_53–60_ showed E4 positivity in all 5 CIN1 associated with HPV-18 DNA (typical staining pattern shown in Fig. 5B) by WTS-PCR, but did not detect E4 in the 1 borderline CIN1 (biopsy 4) that contained HPV-18 DNA by WTS-PCR and for which a section was available. Interestingly, LCM-PCR did not recover HPV DNA from either borderline CIN1 biopsy (biopsy 4 and 5), which suggests that HPV-18 may not be causative. Unfortunately, the limited availability of sections precluded R18E4_53–60_ staining in the second borderline CIN1 (biopsy 5), although the positive E4 signal with R58E4_23–30_ and absence of staining with TVG405 did reveal active HPV-58 expression in regions not sampled by LCM. The CIN1/2 biopsy (biopsy 19) was E4-negative by IHC with R18E4_53–60_, and was typed as HPV-31 by LCM-PCR. Among 6 HPV-18 DNA CIN2 biopsies by WTS-PCR, 3 showed positive HPV-18 E4 staining, while 2 biopsies (biopsy 30 and 41) had no HPV-18 DNA by LCM-PCR. All 6 CIN3 biopsies typed as HPV-18 by WTS-PCR were negative for HPV-18-specific E4 expression. Among these, LCM-PCR confirmed biopsy 61 was positive for HPV 18 while biopsy 65 was negative. No other biopsy was positive with the type-specific HPV-18 antibody including those containing HPV-39 (biopsy 6) and -70 (biopsy 43), which are phylogenetically related to HPV-18 and members of the alpha 7 species [21]. The results obtained with the polyspecific antibody TVG405 were entirely compatible with those produced using the type-specific R18E4_53–60_ antibody for all 5 HPV18-associated CIN1 associated with HPV-18, and for the CIN2 and 3 that were tested.

The antibody R58E4_23–30_ showed positivity in 2/2 CIN1 lesions (of which one is a borderline CIN1) typed as HPV-58 by WTS-PCR. The borderline CIN1 (biopsy 5) was negative by LCM-PCR, which may be because a different area was sampled compared to that which was positive by E4 IHC. Only 3 CIN2 biopsies typed as HPV-58 were stained for HPV-58 E4 however 2 were positive, and this result was confirmed by LCM-PCR. Biopsy 34 was used to demonstrate the absence of cross–reactive staining with the type-specific 16 and 18 reagents. Five of the 9 CIN3 lesions typed as HPV-58 by WTS-PCR were positive for HPV-58 E4 (see Fig. 5C for typical staining pattern (case 62)). LCM-PCR confirmed the presence of HPV-58 in 5 of the CIN3 lesions, including 3 of those that were E4-positive. No other section was positive with the type-specific HPV-58 antibody including HPV-16 biopsies (biopsies 35+49), which is phylogenetically related to HPV-58 and a member of the alpha 9 species [21].

**Figure 6:**
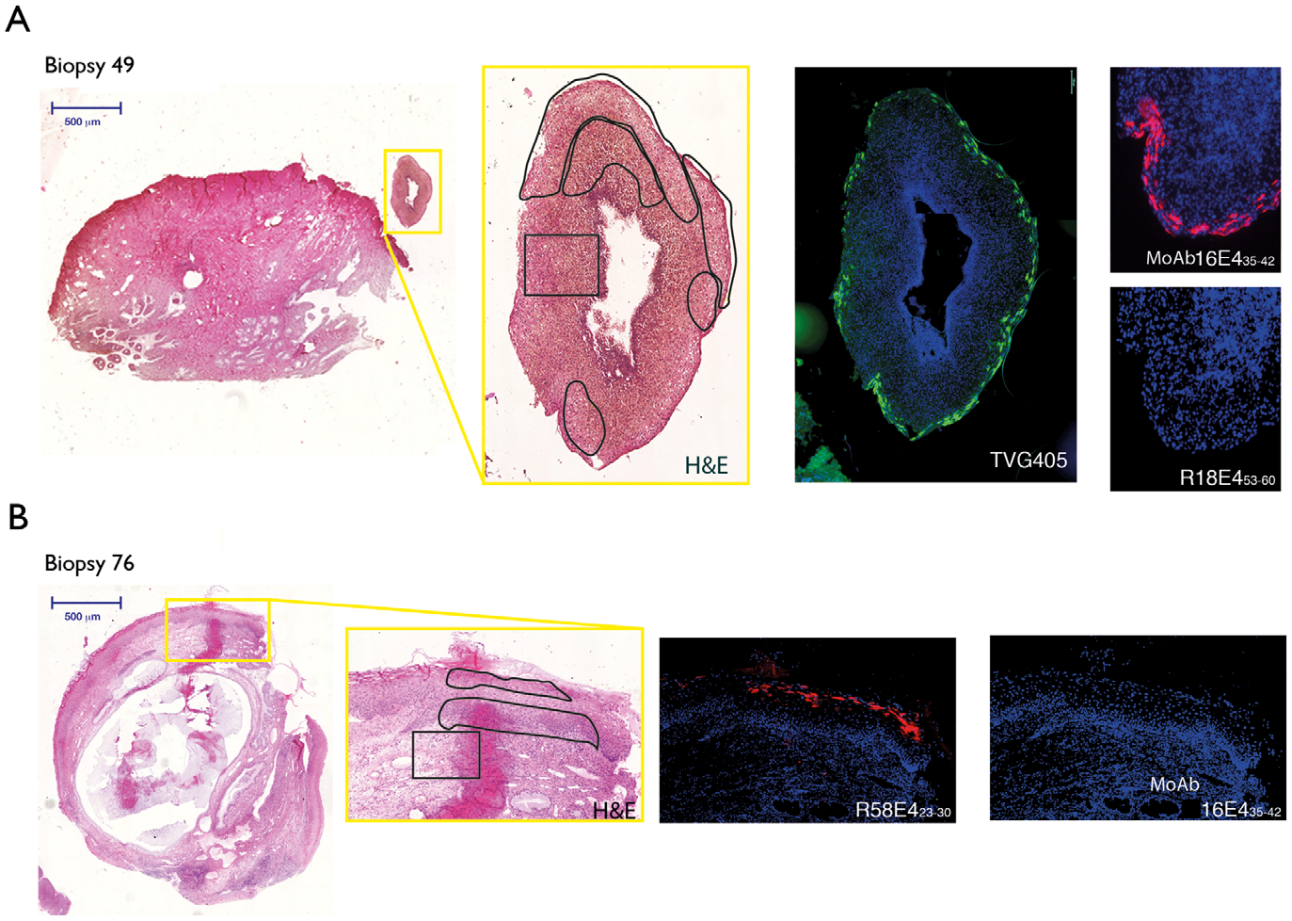
Immunohistochemical staining for HPV E4 in productive cervical lesions caused by different HPV types using MoAb16E4_35–42_, R18E4_53–60_, R58E4_23–30_ or TVG405 antibodies. A) Scan of H&E stained biopsy 49 (genotype HPV-16, 18, 31 by WTS-PCR) with areas of interest (CIN 2) boxed in yellow. Regions analysed by LCM-PCR (genotype HPV-16) are delimitated by black lines. Detection of HPV-16 E4 protein expression on a separate tissue section using MoAb16E4_35–42_ antibody, and confirmed using TVG 405 is shown in an HPV-16-infected region. Antibodies were used together in a double staining regime on the same tissue slice. The HPV18 type-specific antibody (R18E4_53–60_) gave no staining. B) Scan of H&E stained biopsy 76 (genotype HPV-16, 52, 58 by WTS-PCR) with areas of interest (CIN 2) boxed in yellow. Regions analysed by LCM/PCR (genotype HPV-58) are delimitated by black lines on a separate tissue section. The detection of HPV-58 E4 protein using R58E4_23–30_ in an HPV-58-infected region is shown in red following double staining of a single tissue slice. MoAb16E4_35–42_ antibody gave no staining indicating no cross-reactivity.

The patterns of E4 IHC staining and their relation to HPV DNA detection in specific areas of CIN by LCM-PCR were more extensively examined for some biopsies as shown in Fig. 6. Biopsy 49, graded CIN2/3, is HPV-16, -18 and -31 DNA positive as determined by WTS-PCR. E4 IHC showed that HPV-16 E4 protein was expressed, whereas HPV-18 and HPV-31 E4 were not detected (Fig. 6A and data presented in Table 1) while LCM-PCR detected only HPV-16 in the lesion. Therefore, the LCM-PCR and IHC results are in agreement and HPV-16 is assigned as the primary cause of disease apparent in this lesion. For biopsy 76, graded CIN3, and HPV-16, -52 and -58 DNA positive by WTS-PCR, only HPV-58 E4 protein and HPV-58 DNA (by LCM-PCR) were found in the lesion (Fig. 6B), therefore HPV-58 was identified as the causal HPV type. The four HPV DNA negative sections listed in table 1 were all negative with the type-specific E4 antibodies.

### Expression of Type-specific E4 in Graded Tissue Sections Suggests a Role in Molecular Pathology

**Figure 7:**
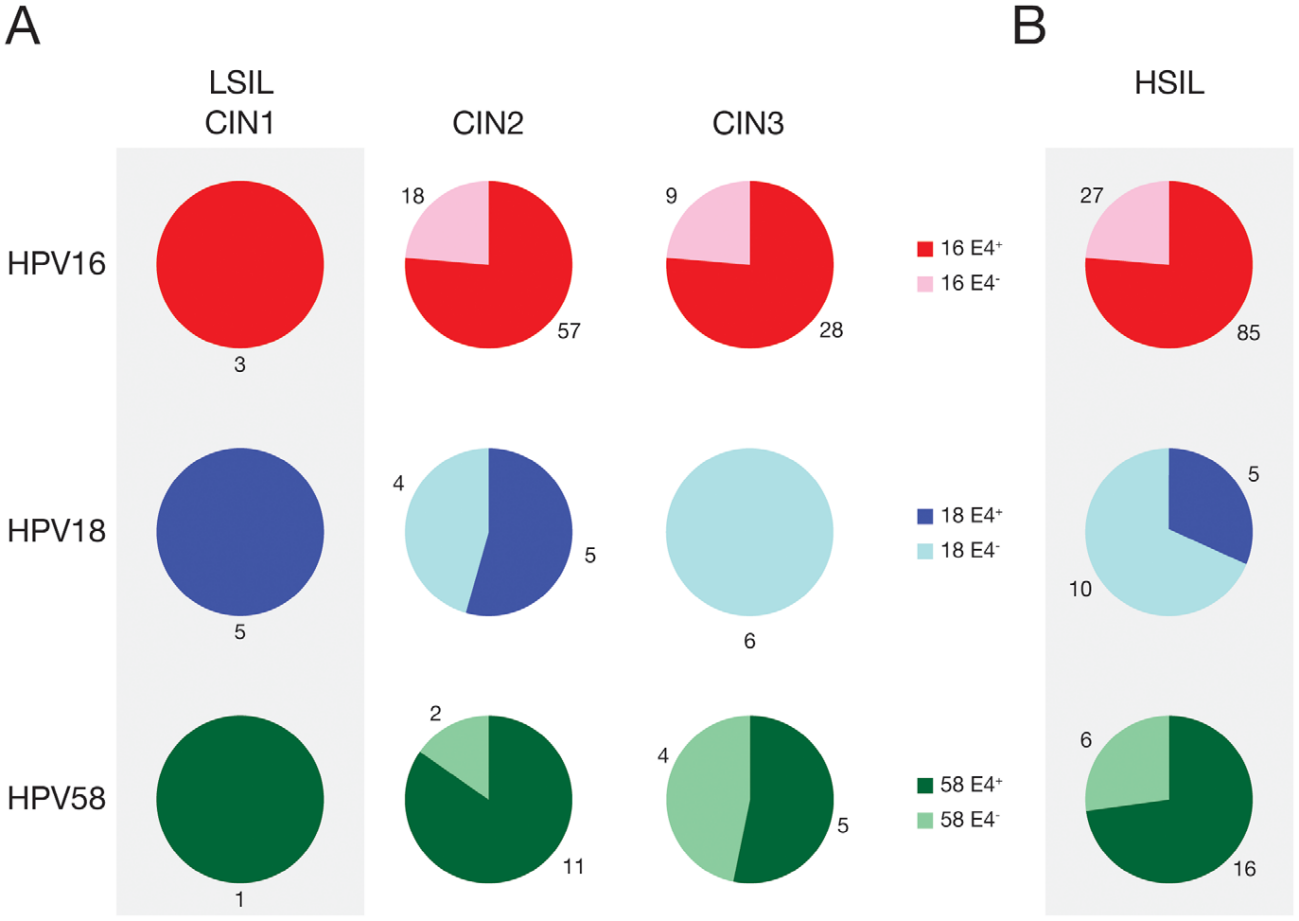
Pie charts showing results of immunohistochemical staining for HPV E4 proteins in productive cervical lesions caused by different HPV types using MoAb16E4_35–42_, R18E4_53–60_ or R58E4_23–30_ antibodies. In (A) cases are stratified according to CIN status. CIN1 is equivalent to LSIL, and in all cases where causality was known, type-specific E4 expression was apparent. Type-specific E4 expression was differentially distributed between lesions with an overall diagnosis of CIN2 or CIN3 depending on causative HPV type. All HPV18 CIN3 lacked E4 expression. In (B), the CIN2 and 3 groupings are pooled to produce the HSIL group. This group could be divided into two categories depending on whether E4 was expressed in the tissue section under examination.

In total, 275 biopsies were stained with type-specific antibodies during the course of this study. Of these, 28 were classified as normal, 2 were graded borderline CIN1, 19 were classified as koilocytic CIN1, 1 was CIN1/2, 166 were CIN2, 2 were CIN2/3 and 57 were CIN3. Classifications were based on the most severe pathology present in the tissue section, although in many cases both low and high-grade disease were found together in the same biopsy. All of the biopsies that could be conclusively graded as CIN (245), and which could reasonably be assigned a causal HPV type either by WTS-PCR (when only one type was present) or by LCM-PCR (when multiple types were present), are shown graphically in Fig. 7. Lesions showing sign of regression (i.e. which had infiltrating lymphocytes) or which were damaged during the IHC procedure were not included, leaving a total of 158 biopsies in total. All biopsies that showed E4 IHC-positive staining were found to contain the relevant HPV type by WTS-PCR typing, with E4-positivity being found in all koilocytic CIN1 irrespective of HPV type (Fig. 7A). In tissue sections showing higher-grade disease, E4 expression was variably present in pockets of epithelial differentiation, and unexpectedly showed different distributions when stratification was made according to HPV type (Fig. 7A). Particularly striking was the absence of E4 expression in any of the HPV18 CIN3 (6/6), which contrasts sharply with the presence of E4 in more than half of the HPV16 (28/37) and 58 CIN3 (5/9). Although the total number of biopsies analysed here was relatively large, HPV16 predominated in the high-grade lesions, and the number of HPV18 and HPV58-associated CIN3 (and also the total number of CIN1) was still however quite small. The CIN2/CIN3 classifications are often considered together as CIN2+ or HSIL (high-grade squamous intraepithelial lesion) in order to distinguish this group, which may require treatment, from the CIN1 or LSIL (low-grade squamous intraepithelial lesion) which generally do not. The expression of E4 effectively divides the CIN2+ group according to its presence or absence (Fig. 7B) and may provide a distinct molecular indicator of life-cycle de-regulation (or possibly even prognosis), that is distinct from the pathology criteria that are currently employed.

## Discussion

Here we show that HPV type-specific anti-E4 antibodies (MoAb16E4_35–42_, R18E4_53–60_ and R58E4_23–30_) can be generated using a short-peptide approach, and that such reagents can be applied to formalin fixed paraffin-embedded clinical tissue sections to identify sites of active infection by specific HPV types. HPV-16, -18 and -58 were chosen for these ‘proof of principle’ studies because of their importance, and because they are representative of the various types of HPV that are associated with cervical cancer. Previously, a synthetic Fab (TVG405), was raised against the full length HPV-16 E4 protein [17], which we now show to cross-react with the E4 proteins of HPV-18, -31, -35 and -45 (Fig. 2). While preparing the type-specific reagents described in this study, we noticed that peptides selected on the basis of amino acid sequence divergence (rather than predicted antigenicity) often elicited a potent type-specific anti-protein response, even when reactivity against the peptide immunogen in ELISA was poor (Fig. 1D). Differences in immune response were also seen between different animal species as well as between inbred animals of the same species (Fig. 1C). Despite these unexpected findings, type-specific anti-E4 responses were achieved for all three HPV types tested. The mouse monoclonal anti-E4 antibody MoAb16E4_35–42_, and the rabbit polyclonal antibodies R18E4_53–60_ and R58E4_23–30_ recognize their specific HPV targets, but do not react with the E4 proteins of even closely related HPV types, including HPV31, HPV 45 and HPV 33.

Cervical HPV infections with multiple oncogenic HPV types are common in sexually active young women [22], but the different HPV types detected in such lesions are not necessarily associated with productive infection or neoplastic transformation. We envisage that type-specific E4 antibodies (such as those described here) will be diagnostically useful for identifying or confirming a causal active infection in lesions where multiple HPV types are present. In this study, E4 staining was often carried out alongside LCM-PCR, with both methodologies producing complementary and entirely compatible results that fit in well with our understanding of HPV disease and its deregulation during cancer progression. Compared to IHC methods however, LCM-PCR is quite labor-intensive and costly, and requires priorevaluation by a pathologist in order to direct the operator to likely sites of disease. Because of this, IHC-based methods of disease-localization are better suited to routine diagnosis, and indeed, first generation biomarkers such as p16, Ki67 and MCM are already available for diagnostic use. As a true virus antigen with a complementary (or inverse) pattern of expression in cervical disease, we suspect that E4 detection will be of value not only in confirming HPV-causality in low-grade lesions, but also in detecting and monitoring the extent and persistence of LSIL, and its possible transition to higher-grade disease. Research on the use of such biomarkers in combination is now very much required.

Our work has also suggested differences in the biology of the three HPV types investigated. There is increasing evidence that the natural history of each oncogenic HPV type is different [23], with HPV-16/18 causing cervical cancer at an earlier age than other HPV types [24]. These two types together cause the majority of cervical cancers, which has lead to them being targeted during the development of HPV prophylactic vaccines [25], [26], [27]. Distinguishing an active infection by these HPV types, from latency or inapparent infections (which may later become active) is important in accurately assessing vaccine performance, and/or when carrying out molecular screening to prevent cervical cancer. HPV-18 is contained within the Alpha 7 group, and is important in adenocarcinoma and other cervical cancers in young women. In contrast, some Alpha 9 HPV types (eg HPV-58), although oncogenic, appear to have a slower and less frequent evolution from infection to invasive cervical cancer [24]. Understanding the molecular basis for differences in natural history between individual oncogenic HPV types is difficult to carry out, with few highly type-specific probes of HPV gene-activity appropriate for clinical research. Indeed, the differences in E4 expression between HPV types during neoplastic progression have not been studied previously. With all three HPV types examined here, it appears that loss of surface epithelial differentation always correlates very closely with the loss of E4 in HSIL lesions. Using the type-specific antibodies however, we have shown differences in E4 expression in CIN3 between HPV-16/58 and HPV-18, with frequent expression of HPV-16 and -58 E4 in CIN3 but not of HPV-18 E4. Variation in the failure to complete a productive life-cycle in CIN3, may provide insight into the to the molecular events that underlie differences in rates of CIN2 and CIN3 and progression to invasive cervical cancer (ICC or regression between oncogenic HPV types. The maintenance of E4 expression in many HPV-58-associated CIN3, and its loss in HPV-18-associated CIN3 is consistent with the different biologies of these two HPV types. HPV-18 is associated with ICC at an earlier age than HPV-58, while HPV-58 is associated with relatively low rates of ICC compared to CIN3 and a later age of development of ICC [22]. The average age of patients with invasive cervical carcinoma caused by HPV-18 (37 years) is also lower than that for HPV-16 [28]. The disruption of the E2 ORF and the deregulation of E7 gene expression that results from integration is considered a major factor in the development of many cervical cancers. Viral genome integration can also lead to the loss of negative regulatory elements that normally limit the abundance of E6 and E7 mRNAs during normal productive infection. Interestingly, the E4 proteins of both HPV-16 and HPV-18 induce G2 arrest in cervical epithelial cells [29], [30], [31], and their expression in the epithelium is not compatible with continued cell proliferation. As overlapping genes, the disruption of the E4 ORF often accompanies the disruption of the E2 ORF, and this may be an additional predisposing factor in neoplastic progression. The absence of E4 expression in HPV-18 associated CIN3 may facilitate, or even be necessary for E6/E7-mediated cell proliferation throughout the epithelium. Interestingly, other groups [32] have reported HPV 18 integration in women with CIN3 and observed that cytologic changes detected after HPV-18 infection underestimate the severity of the underlying histologic abnormalities. When taken together, these results indicate a need to improve the efficiency of cervical screening, particularly with regards to HPV-18-associated abnormalities where E4 staining may help discriminate between the LSIL and HSIL groups. A wider panel of E4 antibodies would facilitate clinical research into the different patterns of disease progression that are apparent when different HPV types are compared.

Since the HPV life cycle and E4 expression are linked to epithelial differentiation, a differentiating organotypic raft culture model was used here to allow us to recreate the productive cycle of individual HPV types [10]. This is the first reconstruction of the HPV-18 productive cycle other than in primary cells, and the first report of *in vitro* HPV-58 life cycle reconstruction. The three recognizable phases of the virus life cycle; cell proliferation, the appearance of E4 (genome amplification) and L1 expression (genome packaging) were apparent using antibodies to MCM, HPV E4 and HPV L1, respectively, in rafts infected with HPV-16, 18 or 58. Subsequent staining with the anti-E4 peptide antibodies detected E4 in differentiating epithelial cells and accurately distinguished between HPV types. In this study, the HPV rafts served as an important tool for optimizing the staining procedures for the type-specific anti-E4 antibodies, and were used as positive controls when examining clinical tissue sections. The approach may be generally useful for the evaluation of HPV biomarkers, especially for HPV types that are found infrequently in the population.

The results of our work are broadly consistent with the previous more limited studies of E4 expression using poly-specific antibodies [7], [17]. HPV-16, -18 and -58 infections showed expression of E4 in all koilocytotic CIN1 samples where causality could be conclusively attributed (i.e. single typed WTS-PCR or typed by LCM PCR). The E4-negative CIN1 and borderline CIN1 may represent non-viral lesions, and in such cases, viral markers such as E4 may be particularly valuable clinically. The E4-positive borderline CIN1 was HPV-58 E4 positive in a different area to that sampled by LCM, and highlights a limitation of the LCM approach, which cannot feasibly sample and attribute causality to all areas of disease within a tissue section. Of the undamaged CIN2 biopses that were convincingly attributed to HPV 16 by a combination of WTS-PCR and LCM-PCR, 61.5% (8/13, see Table 1) expressed E4. When all HPV16-associated CIN2 are considered (Fig. 7), (57/75) 76% were found to express E4. Of the CIN2 biopsies convincingly attributed to HPV-18 (3/4), 75% expressed E4 (Table 1), and of the CIN2 biopsies convincingly attributed to HPV-58 (2/3), 66% expressed E4 if appropriately stained. Analysis of the larger grouping (which were not always typed by both WTS-PCR and LCM-PCR) revealed a similar distribution (Fig.7). None of the HPV-18 attributed CIN3 biopsies expressed E4 (0/5, Table 1 (0/6 from Fig. 7)) while (5/9) 55.6% (Table 1 and Fig.7) of the HPV-58 attributed CIN3 biopsies expressed E4. E4-positivity in HPV16-attributed CIN3 biopsies was (8/14) 51.7% (Table 1) and (28/37) 76% in the more extensive analysis shown in Fig. 7. Histological review of the CIN2 and CIN3 sections without E4 expression revealed limited or no surface epithelial differentiation. Limited sample numbers of CIN1 and HPV-18, -58 associated CIN2 and CIN3 may affect the specific results, but the underlying trends are similar to those observed and published previously [7], [17].

All of the established biomarkers in current use have some limitations. For example, antibodies to MCM and Ki-67, which are used as surrogate markers of E6 and E7 expression in HPV-associated CIN, will also identify cells that are proliferating during normal metaplasia or wound healing. These markers are also found in replication competent (but non-dividing) cells that are supporting viral genome amplification, even in low-grade lesions caused by low-risk HPV types. Similarly p16 may be detected in senescent cells in the upper layers of the epithelium in high-risk HPV-associated CIN, as well as in E6/E7-expressing cells of the basal and parabasal layers [5], [7], [33]. To provide additional clarity, these markers (e.g. p16 and Ki67), can be used together to improve clarity, or be combined with markers that provide additional information, such as E4 or fluorescence in situ hybridization (FISH) which detect the onset of genome amplification, or L1 which detects the onset of virus assembly. E4 expression is restricted to the superficial differentiated squamous cells (Fig. 3A) and there is an inverse relation between transformation as shown by CIN grade and E4 expression. The fact that the E4 protein is expressed in all HPV-associated low-grade lesions, but in only a subset of high-grade lesions indicates the limitation of E4 antibodies when used as a single biomarker. It appears therefore, the detection of HPV E4 expression by IHC should ideally be combined with other markers, such as MCM or p16, which have complementary expression patterns. As the viral coat protein L1 is only expressed in a subset of additionally differentiated cells that already contain E4, it would appear that L1 is less suitable for the detection of HPV activity in CIN than E4. Indeed, our ongoing studies suggest that a primary stain combining the E4 marker with MCM greatly improves lesion detection as well as our understanding of disease status (ZW, HG, JD personal communication). These studies are now making use of a newly generated pan-E4 monoclonal antibody (FH1.1), which is capable of detecting at least 13 types of high-risk HPV E4 proteins in clinical tissue biopsies. Using a pan- E4 antibody for initial diagnosis should allow identification of LSIL and productive subsets of HSIL, which may be associated with more frequent regression rather than progression to cancer. We envision the use of type-specific antibodies in a second stain, possibly combined with DNA typing, on E4 positive and therefore productive lesions with multiple HPV infection to establish causative type. This could permit the development of HPV E4 approaches to improve cervical screening and patient management of CIN for the era of HPV vaccination. Although further work is required to implement these proposed ideas, our work to date has shown that type-specific E4 antibodies can be generated and used to help locate areas of active infection when a particular HPV type is detected at the level of DNA. Such type-specific antibodies may also be used to establish the cause of low-grade, and a subset of high-grade disease (Fig.7), in situations where multiple HPV types are present.
